# LASP1 Induces Epithelial-Mesenchymal Transition in Lung Cancer through the TGF-*β*1/Smad/Snail Pathway

**DOI:** 10.1155/2021/5277409

**Published:** 2021-12-06

**Authors:** Qingming Xue, Hong Jiang, Jinjie Wang, Dongshan Wei

**Affiliations:** ^1^Department of Thoracic Surgery, Affiliated Hangzhou First People's Hospital, Zhejiang University School of Medicine, Hangzhou, China; ^2^Department of Pathology, Affiliated Hangzhou First People's Hospital, Zhejiang University School of Medicine, Hangzhou, China

## Abstract

**Background:**

LIM and SH3 domain protein 1 (LASP1), highly expressed in a variety of tumors, is considered as a novel tumor metastasis biomarker. However, it is unknown which signaling pathway works and how the signal transduces into cell nucleus to drive tumor progression by LASP1. The aim of this study is to explore the essential role of LASP1 in TGF-*β*1-induced epithelial-mesenchymal transition (EMT) in lung cancer cells.

**Methods:**

The gene and protein levels of LASP-1 were successfully silenced or overexpressed by LASP-1 shRNA lentivirus or pcDNA in TGF-*β*1-treated lung cancer cell lines, respectively. Then, the cells were developed EMT by TGF-*β*1. The cell abilities of invasion, migration, and proliferation were measured using Transwell invasion assay, wound healing assay, and MTT assay, respectively. Western blotting was used to observe the protein levels of EMT-associated molecules, including N-cadherin, vimentin, and E-cadherin, and the key molecules in the TGF-*β*1/Smad/Snail signaling pathway, including pSmad2 and Smad2, pSmad3 and Smad3, and Smad7 in cell lysates, as well as Snail1, pSmad2, and pSmad3 in the nucleus.

**Results:**

TGF-*β*1 induced higher LASP1 expression. LASP1 silence and overexpression blunted or promoted cell invasion, migration, and proliferation upon TGF-*β*1 stimulation. LASP1 also regulated the expression of vimentin, N-cadherin, and E-cadherin in TGF-*β*1-treated cells. Activity of key Smad proteins (pSmad2 and pSmad3) and protein level of Smad7 were markedly regulated through LASP1. Furthermore, LASP1 affected the nuclear localizations of pSmad2, pSmad3, and Snail1.

**Conclusion:**

This study reveals that LASP1 regulates the TGF-*β*1/Smad/Snail signaling pathway and EMT markers and features, involving in key signal molecules and their nuclear levels. Therefore, LASP1 might be a drug target in lung cancer.

## 1. Introduction

Targeted therapy and new targets provide new hope for the treatment of NSCLC lung cancer [1]. In 1995, LIM and SH3 domain protein 1 (LASP1) was firstly identified with high gene expression in malignant cancer tissues by Tomasetto [2, 3]. In recent two decades, as the number of reports increased, the understanding of the physiological and pathological functions of LASP1 has been deepened and widened, especially in the view that LASP1 has the potential to become a cancer biomarker. In 2015, Orth et al. [4] suggested LASP1 as the versatile structural, signaling, and biomarker protein and indicated the importance of LASP1 in cancer pathology. More and more evidence [5] supports LASP1 as a novel tumor metastasis protein.

In lung cancer metastasis, the role of LASP1 has only recently been explored and needs to be further studied. In 2016, Zheng et al. [6], for the first time, reported that LASP1 is highly expressed in human non-small-cell lung cancer (NSCLC) and suggested LASP1 as an independent prognostic factor for NSCLC. Fahrmann et al. [7] also recognized LASP1 as the negative predictor of survival. In terms of the mechanism of LASP1 in NSCLC, studies have been focused on the upstream molecules regulating LASP1, including SOX9 [8], microRNA-29a [9], and microRNA-203 [6]. Only Zhang et al. [10] have reported that LASP1 induces phosphorylation of the FAK-AKT signaling pathway to promote the malignant phenotype of NSCLC. In general, the research on the mechanism of LASP1 regulating lung cancer is still in its infancy. The downstream molecules regulated by LASP1 also need to be supplemented.

Epithelial-mesenchymal transition (EMT) is widely considered to contribute to the malignancy of lung cancer [11, 12], involving in the invasion, proliferation, adhesion, and metastasis of lung carcinoma cells [13–15]. The correlation between LASP1 and TGF-*β*1-mediated EMT has been little studied in lung cancer, although it has been reported in other cancers. Zhong et al. [16] reported that LASP1 induces glioma growth and invasion through EMT. Wang et al. [17] and Niu et al. [18] found that LASP1 plays the central role in TGF-*β*1-mediated EMT in colorectal cancer. However, the relationship of LASP1 with TGF-*β*1-mediated EMT in lung cancer has been rarely studied.

TGF-*β*1 regulates EMT via transcription and posttranscription pathways [19], such as the TGF-*β*1/Smad/Snail signaling pathway [20, 21]. A variety of transcription factors are involved in EMT [22, 23]. The central action in EMT is E-cadherin loss [24], which is regulated by Snail proteins at the transcriptional level. Duvall-Noelle et al. [25] found that LASP1 directly binds to and stabilizes Snail1 in breast cancer cell lines. We speculated that, in lung cancer, LASP1 might be involved in the process of TGF-*β*1-mediated EMT and might affect the relevant transcriptional molecules in cell nuclei. However, still more studies are needed to verify the role of LASP1, as well as the complete and coherent participating members in the TGF-*β*1-mediated EMT signaling pathway.

In this study, it is aimed to elucidate the role of LASP1 in the proliferation, invasion, migration, EMT, and nuclear expression of key molecules in lung cancer cell lines upon TGF-*β*1 stimulation.

## 2. Materials and Methods

### 2.1. Cell Lines

Two human lung cancer cell lines, A549 and SK-MES-1, were purchased from Shanghai Institutes for Biological Sciences (SIBS, Shanghai, China) and cultured in DMEM (Invitrogen, Carlsbad, USA) with 10% FBS (Invitrogen) at 37°C and 5% CO_2_.

### 2.2. Immunohistochemical (IHC) Staining

12 pairs of NSCLS tissues and the matched adjacent nonmalignant tissues were collected and embedded by paraffin. Then, the tissue sections were cut by using a microtome at 4 *μ*m. The slides were dewaxed, rehydrated, and retrieved in citric acid buffer (pH6.0) by using a microwave oven. Subsequently, the tissue slides were blocked by normal goat serum and then were incubated with a 1 : 50 dilution of anti-LASP1 antibody (Boster, Wuhan, China) for 2 h at 37°C. After washing with PBS, the slides were incubated with secondary antibody for 1 h at 37°C, followed by washing with PBS and staining with a DAB Detection Kit (Boster). Nuclei were counterstained with hematoxylin. The images were obtained by using a microscope (Nikon, Japan).The semiquantitative analysis for positively stained cells was performed by Image-Pro Plus software (v6.0) to express results as average optical density (AOD) at a magnification of ×400.

### 2.3. Plasmids, Small-Interfering RNAs (siRNAs), and Small Hairpin RNA (shRNA)

The overexpression plasmid was generated by inserting LASP1 cDNA into the pcDNA3.1 vector, and sequencing was confirmed by GenePharma (Shanghai, China). Several siRNA duplexes (Table S1, supplementary data) were synthesized from GenePharma to reduce human LASP1 (NM_001271608.1), and we selected the best one by RT-qPCR (duplex 2, Figure S1, supplementary data), which is in accordance with the reported sequence by Traenka et al. [26]. Scramble siRNA was also obtained with the sequence of 5′-CCTAAGGTTAAGTCGCCCTCG-3′. shRNA targeting LASP1 or scrambled shRNA was prepared by GenePharma based on the siRNA sequences and were used to construct the shRNA lentivirus, respectively.

### 2.4. LASP1 Overexpression, Knockdown, and Grouping

Two human lung cancer cell lines were transfected with either the empty vector control or LASP1 expression vector using Lipofectamine 3000 (Invitrogen) following the manufacturer's protocol. For knockdown assays, two cell lines with 70% confluence were infected by lentivirus expressing LASP1 shRNA or scrambled shRNA at a multiplicity of infections (MOI) of 40 virus particles per cell. In knockdown and overexpression assays, 2 ng/ml recombinant TGF-*β*1 (Sigma, MA, USA) was added to produce EMT stimulation.

There were six groups in this study. Two groups were treated with 2 ng/ml TGF-*β*1 and infected with scramble shRNA or LASP1 shRNA and then named as SC shRNA and LASP1 shRNA, respectively. Two groups were treated with 2 ng/ml TGF-*β*1 and transfected with the empty vector or LASP1 expression vector and then named as pcDNA and pcDNA-LASP1, respectively. Cell groups were used as controls in the presence and absence of TGF-*β*1 treatment and then named as TGF-*β*1 and cells, respectively.

### 2.5. Quantitative Real-Time PCR (qRT-PCR)

After 48 h infection and TGF-*β*1 treatment, total RNA was extracted from the cells using Trizol reagent (Invitrogen). Then, RNA concentration was detected using NanoDrop. cDNA was reversely transcripted from 1 *μ*g of total RNA of each sample using random primers. The mRNA levels were quantified using fluorescent quantitative PCR amplification with the following condition: predenaturation at 95°C for 4 min followed by 40 cycles (94°C × 30 sec for denaturation, 57°C × 30 sec for annealing, and 72°C × 2 min for extension) and finally extension at 72°C × 10 min. Primer sequences for LASP1 and GAPDH cDNA were as follows: the forward of LASP1 5′-GTGTATCCCACGGAGAAGGT-3′ and the reverse of LASP1 5′-TGCCACTACGCTGAAACCT-3; the forward of GAPDH5′-ACAACTTTGGTATCGTGGAAGG-3′ and the reverse of GAPDH5′-GCCATCACGCCACAGTTTC-3′.

### 2.6. Transwell Matrigel Invasive Assay

Human lung cancer cell invasion was assessed by using a chamber culture system (8 *μ*m pore size). The cells were coinfected with TGF-*β*1 and LASP1 shRNA or TGF-*β*1 and LASP1 pcDNA for 24 h. Afterwards, the cells were inoculated at 2 × 10^4^ cells/100 *μ*l into the upper Matrigel-coated chamber in a medium containing 1% FBS per transwell, and 700 *μ*l medium with 2 ng/ml TGF-*β*1 was added into the lower chamber. 48 h later, cells in the upper side of the chamber were carefully wiped out with a cotton swab. Then, cells in the bottom side were fixed with 4% paraformaldehyde and stained with leucocrystal violet.

### 2.7. Wound Healing Assay

After 24 h coinfection with TGF-*β*1 and LASP1 shRNA or TGF-*β*1 and LASP1 pcDNA in a 6-well plate, the wounds were created by a pipette tip in 70% confluence cells. Then, cells were rinsed with PBS to remove floating cells and debris. After 24 h incubation with the medium containing 2 ng/ml TGF-*β*1 and 10% FBS, the photographs were taken by using a microscope.

### 2.8. MTT Assay

After 24 h coinfection with TGF-*β*1 and LASP1 shRNA or TGF-*β*1 and LASP1 pcDNA, the cells were trypsinized and seeded in 96-well plates with a density of 2000/well. After 12 h∼72 h culture, cell growth was determined by cell medium containing MTT (final concentration: 0.5 mg/ml) in each well for 2 h incubation. Then, 490 nm absorbance values were read.

### 2.9. Western Blotting

After coinfection with TGF-*β*1 and LASP1 shRNA or TGF-*β*1 and LASP1 pcDNA for 48 h, the whole-cell fractions and nuclear fractions from the two lung cancer cell lines were prepared using the nuclear protein extraction kit (Beyotime, Shanghai, China) following the manufacturer's instruction. The total protein contents were measured by Coomassie brilliant blue assay. The total proteins binding with SDS were electrophoresed on 10% SDS-PAGE and transferred to PVDF membranes. The membranes were immune-blotted with the primary antibody overnight at 4°C and subsequently immune-blotted with secondary antibody (Boster) for 2 h at room temperature. Blots were visualized using the ECL method (Boster). Primary antibodies of LASP1 (#8636, 1 : 1000), N-cadherin (#4061, 1 : 1000), E-cadherin (#3195, 1 : 1000), vimentin (#5741, 1 : 1000), pSmad2 (#3108, 1 : 1000), Smad2 (#3122, 1 : 1000), pSmad3 (#9520, 1 : 500), Smad3 (#9523, 1 : 500), Snail1 (#3879, 1 : 1000), GAPDH (#2118, 1 : 1000), *α*-tubulin (#2144, 1 : 1000), and Lamin B (#13435, 1 : 500) were purchased from Cell Signaling Technology (Massachusetts, USA). Smad7 primary antibody (#25840-1-AP, 1 : 500) was purchased from Proteintech (Illinois, USA).

### 2.10. Statistical Analysis

We used one-way analysis of variance (ANOVA) to compare the differences, followed by the LSD-Q test for comparison between groups. SPSS program (v.13.0, SPSS, USA) was used to perform the statistical analysis. Results were presented as mean ± SD with *p* value less than 0.05 as statistical significance.

## 3. Results

### 3.1. Expression of LASP1 in NSCLC and Nonmalignant Adjacent Tissue

**Figure 1:**
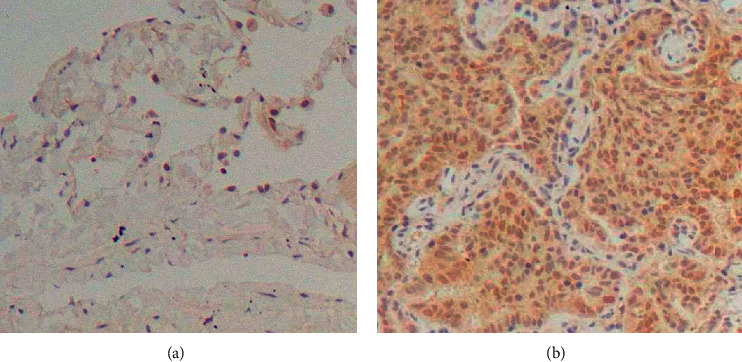
The expressions of LASP1 in human nonmalignant adjacent tissues and lung adenocarcinoma tissues were detected by immunohistochemical staining (magnification, ×400). (a) Adjacent. (b) Adenocarcinoma.

Initially, LASP1 expression was determined in 12 pairs of NSCLC and nonmalignant adjacent tissue specimens using immunohistochemistry staining. To quantify protein expression, semiquantitative analysis of average optical density values was performed and showed that LASP1 expressed significantly higher in lung cancer tissues than the matched adjacent control (Figure 1 and Table 1).

### 3.2. LASP1 Knockdown and Overexpression in TGF-*β*1-Treated Human Lung Cancer Cell Lines

**Figure 2:**
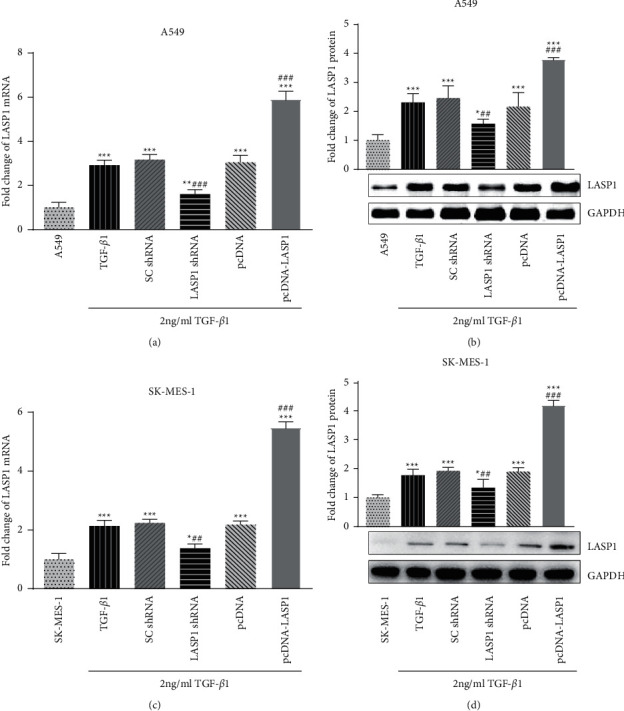
(a–d) LASP1 knockdown and overexpression efficiency in TGF-*β*1-treated A549 and SK-MES-1 cells. After coinfection with 2 ng/ml TGF-*β*1 and lentivirus containing LASP1 shRNA or 2 ng/ml TGF-*β*1 and pcDNA-LASP1, the cells were lysated to detect mRNA and protein levels of LASP1. Fold change mRNA expression levels of LASP1 were quantified by RT-qPCR in A549 (a) and SK-MES-1 (c) cells. Western blot confirmed the change of LASP1 protein levels in A549 (b) and SK-MES-1 (d) cells. All quantitative amounts were normalized against GAPDH expression and shown as mean ± SD, *n* = 6. ^*∗*^*p* < 0.05 and ^*∗∗∗*^*p* < 0.001*vs.* the cell alone group without TGF-*β*1 treatment, and ^#^*p* < 0.05, ^##^*p* < 0.01, and ^###^*p* < 0.01*vs.* the TGF-*β*1-treated noninfection group.

As shown in Figure 2, LASP1 mRNA and protein were expressed highly by TGF-*β*1 stimulation in two cell lines (*p* < 0.05). During coinfection with 2 ng/ml TGF-*β*1 and LASP1 shRNA, LASP1 mRNA (Figures 2(a) and 2(c)) and protein **(**Figures 2(b) and 2(d)) levels were significantly reduced, compared to the TGF-*β*1-treated noninfection group (TGF-*β*1) (*p* < 0.05). Also, during coinfection with 2 ng/ml TGF-*β*1 and LASP1 pcDNA, LASP1 mRNA (Figures 2(a) and 2(c)) and protein (Figures 2(b) and 2(d)) levels were all significantly increased compared to the TGF-*β*1 group (*p* < 0.05). The differences among groups of TGF-*β*1, SC shRNA, and pcDNA were not obvious (*p* > 0.05). These results proved that although TGF-*β*1 stimulated the increased expression of LASP1, the lentivirus containing LASP1 shRNA still inhibited the induced LASP1, whereas LASP1 pcDNA enhanced the further expression. The cell models could be used for the next experiments. In addition, we observed a higher increase by LASP1 pcDNA in SK-MES-1 than in A549 cells, which might be due to the relatively lower LASP1 expression in SK-MES-1 than in A549.

### 3.3. LASP1 Overexpression Enhances Whereas Knockdown Suppresses EMT-Like Features in TGF-*β*1-Treated Lung Cancer Cell Lines

**Figure 3:**
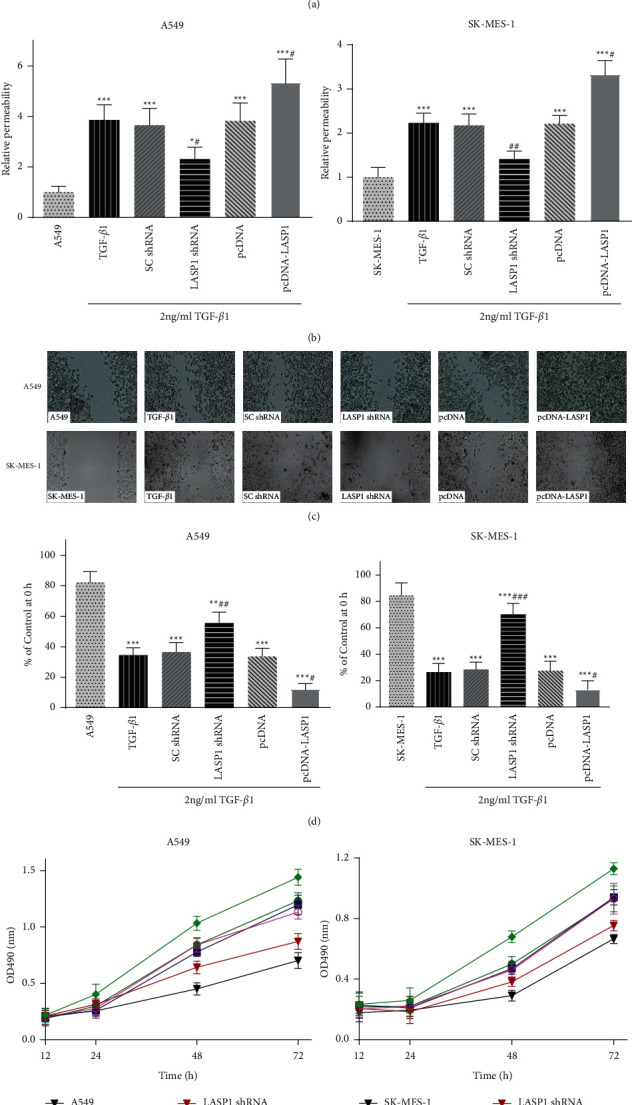
(a–e) Effect of LASP1 on the pathological features of TGF-*β*1-treated A549 and SK-MES-1. (a) The representative images of transwell assays showed the inhibitory or enhanced effect of LASP1 silence or overexpression under TGF-*β*1-induced invasion, respectively. (b) The quantification showed the relative permeability compared to the cell alone group without TGF-*β*1 treatment. (c) The representative images of wound healing assays showed the inhibitory or enhanced effect of LASP1 silence or overexpression under TGF-*β*1-induced migration. (d) The quantification showed the relative migration. (e) LASP1 silence or overexpression had an inhibitory or enhanced effect on lung cancer cell's proliferation until to 72 h under TGF-*β*1 treatment, respectively. Data were shown as mean ± SD, *n* = 3. ^*∗*^*p* < 0.05, ^*∗∗*^*p* < 0.01, and ^*∗∗∗*^*p* < 0.001*vs.* the cell alone group without TGF-*β*1 treatment, and ^#^*p* < 0.05, ^##^*p* < 0.01, and ^###^*p* < 0.001*vs.* the TGF-*β*1-treated noninfection group (TGF-*β*1).

To assess the effect of LASP1 on lung cancer cells, the proliferation effect of LASP1 was analyzed in the presence and absence of TGF-*β*1 treatment. The proliferation result indicated that LASP1 genetic modification in the presence of TGF-*β*1 affected cell growth much more than in the absence of TGF-*β*1 both at 48 h and 72 h (Figure S2, supplementary data). Thus, the role of LASP1 in TGF-*β*1-induced EMT in lung cancer cells was investigated. Transwell invasive assay, wound healing assay, and MTT assay were used to verify the invasion, migration, and proliferation, respectively, by LASP1 overexpression or knockdown. As expected, TGF-*β*1 could significantly increase the ability of invasion (Figures 3(a) and 3(b)), migration (Figure 3(c) and 3(d)), and proliferation (Figure 3(e)) of both A549 and SK-MES-1 cells compared to the cell alone group. Cancer cell invasion ability was significantly increased by TGF-*β*1, whereas it was attenuated by LASP1 silence or further enhanced by overexpression (Figures 3(a) and 3(b)). In Figures 3(c) and 3(d), LASP1 knockdown also notably expanded the narrow distance under TGF-*β*1 stimulation, and LASP1 overexpression promoted cell migration. In addition, LASP1 shRNA induced the inhibitions of proliferation when compared to the SC shRNA group, whereas LASP1 pcDNA further increased the proliferation compared to the pcDNA group (Figure 3(e)).

### 3.4. LASP1 Regulates EMT Hallmark Proteins under TGF-*β*1 Treatment

**Figure 4:**
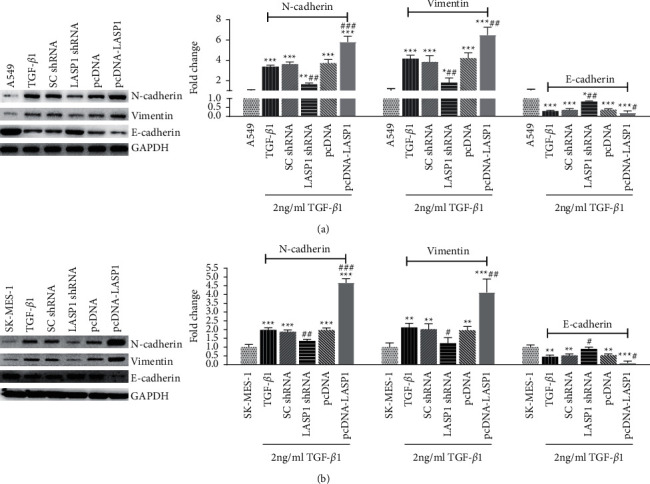
(a-b) LASP1 regulated EMT-related markers expression in A549 (a) and SK-MES-1 (b) cell lines under TGF-*β*1 treatment. After 48 h coinfection with TGF-*β*1 accompanying LASP1 knockdown or overexpression, cell lysates were detected by western blot for EMT-related marker expression, including N-cadherin, vimentin, and E-cadherin. GAPDH was used as reference control. Data were shown as mean ± SD, *n* = 3. ^*∗*^*p* < 0.05, ^*∗∗*^*p* < 0.01, and ^*∗∗∗*^*p* < 0.001*vs.* the cell alone group without TGF-*β*1 treatment, and ^#^*p* < 0.05, ^##^*p* < 0.01, and ^###^*p* < 0.001*vs.* the TGF-*β*1-treated noninfection group (TGF-*β*1).

Because TGF-*β*1 is a prototypical cytokine for EMT induction, we decided to investigate the changes on EMT-related markers by LASP1 silence or overexpression under TGF-*β*1 treatment. As shown in Figure 4, western blot results in both cell lines showed that expressions of mesenchymal markers (N-cadherin and vimentin) were decreased notably by LASP1 knockdown (*p* < 0.05) although under TGF-*β*1 stimulation, and expressions of epithelial marker (E-cadherin) were increased notably (*p* < 0.05), compared to the TGF-*β*1 control group. However, LASP1 overexpression accumulated more N-cadherin and vimentin and less E-cadherin than the TGF-*β*1 control group (*p* < 0.05).

### 3.5. LASP1 Regulates pSmad2, pSmad3, and Smad7 in the TGF-*β*1 Signaling Pathway

**Figure 5:**
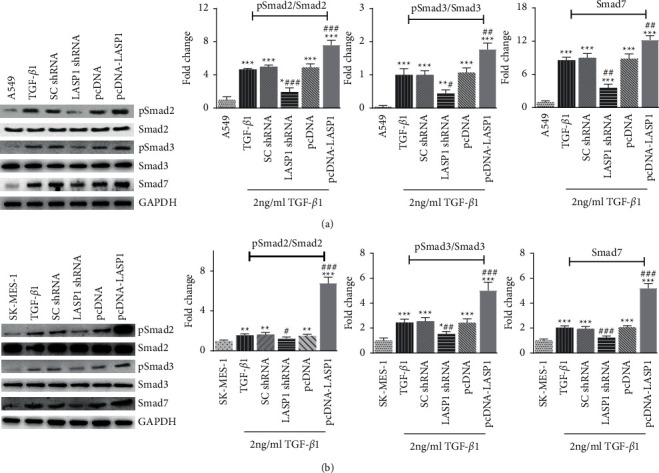
(a-b) LASP1 effect on key molecules in the TGF-*β*1/Smad/Snail signaling pathway in A549 (a) and SK-MES-1 (b) cell lines under TGF-*β*1 treatment. After 48 h coinfection with TGF-*β*1 accompanying LASP1 knockdown or overexpression, the cell lysates were detected for protein levels of pSmad2, Smad2, pSmad3, Smad3, and Smad7 by western blot. Ratios of pSmad2/Smad2, pSmad3/Smad3 and Smad7/GAPDH were presented by gray intensity analysis. GAPDH was used as reference control. Data were shown as mean ± SD, *n* = 3. ^*∗*^*p* < 0.05, ^*∗∗*^*p* < 0.01, and ^*∗∗∗*^*p* < 0.001*vs.* the cell alone group without TGF-*β*1 treatment, and ^#^*p* < 0.05, ^##^*p* < 0.01, and ^###^*p* < 0.001*vs.* the TGF-*β*1-treated noninfection group (TGF-*β*1).

Smad proteins have the key role of transducing TGF-*β*1-induced signals from the cytoplasm to the nucleus. Smad2 and Smad3 are central molecules in EMT induction by TGF-*β*1, and the representative role of their phosphorylated forms (pSmad2 and pSmad3, respectively) has been highlighted in recent years [27]. Smad7 is an antagonistic Smad protein [28]. Due to the actions of LASP1 in the EMT process, we explored its influence to the Smad family including various Smad subtypes. As shown in Figure 5, results of western blots showed that TGF-*β*1-increased phosphorylation of Smad2 and Smad3 was reversed by LASP1 knockdown treatment compared to the TGF-*β*1 group (*p* < 0.05) in both cell lines, whereas LASP1 overexpression enhanced the phosphorylation. However, total Smad2 and Smad3 levels were not impacted in all groups (*p* > 0.05). These results indicated that LASP1 induced dynamic activities of TGF-*β*1/Smad signaling, not protein levels of Smad2 or Smad3, which was due to the unaffected levels of total Smad2 and total Smad3 under TGF-*β*1 treatment (Figures 5(a) and 5(b)). In Figure 5, it can be seen that Smad7 was significantly increased by TGF-*β*1 treatment (*p* < 0.05), whereas LASP1 knockdown or overexpression retarded or aggregated Smad7, respectively (*p* < 0.05). In addition, the fold increases of pSmad2, pSmad3, and Smad7 by LASP1 pcDNA in SK-MES-1 were all higher than those in A549 (Figure 5), which might be correlated with the higher increase of LASP1 in SK-MES-1 than in A549 (Figure 2).

### 3.6. LASP1 Involves in the Regulation of Nuclear Levels of pSmad2, pSmad3, and Snail1

**Figure 6:**
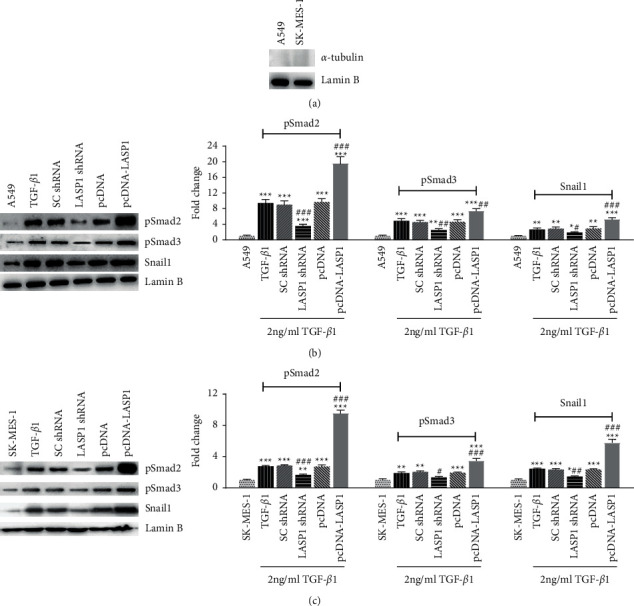
(a–c) LASP1 effect on nuclear levels of key molecules in the TGF-*β*1/Smad/Snail signaling pathway in A549 and SK-MES-1 cell lines under TGF-*β*1 treatment. After 48 h coinfection with TGF-*β*1 accompanying LASP1 knockdown or overexpression, the nuclear fractions were detected for protein levels of pSmad2, pSmad3, and Snail1 by western blot. (a) *α*-Tubulin (cytoplasmic marker) and Lamin B (nuclear marker) demonstrated the purified nuclear extracts. The representative images of western blot and the quantification normalized with (b) Lamin B in A549 and (c) SK-MES-1 cells. Data were shown as mean ± SD, *n* = 3. ^*∗∗*^*p* < 0.01 and ^*∗∗∗*^*p* < 0.001*vs*. the cell alone group without TGF-*β*1 treatment, and ^#^*p* < 0.05, ^##^*p* < 0.01, and ^###^*p* < 0.001*vs.* the TGF-*β*1-treated noninfection group (TGF-*β*1).

Since the EMT-related downstream molecules can be regulated at the transcriptional level and nuclear translocation occurs in the TGF-*β*1/Smad/Snail1 signaling pathway, we next investigated the nuclear levels of pSmad2, pSmad3, and Snail1. The lack of *α*-tubulin (cytoplasmic marker) and the presence of Lamin B demonstrated the purified nuclear extracts in these preparations (Figure 6(a)). Compared to the TGF-*β*1 group, LASP1 silence and overexpression caused a significant decrease and increase of both pSmad2 and pSmad3, respectively (Figures 6(b) and 6(c)) in nuclei (*p* < 0.05). Meanwhile, nuclear Snail1 was also changed with a similar trend (Figures 6(b) and 6(c)) (*p* < 0.05). These results indicated that LASP1 regulated the nuclear levels of pSmad2, pSmad3, and Snail1. In addition, we also observed the higher-fold increases by LASP1 pcDNA in SK-MES-1 than in A549 (Figures 6(b) and 6(c)) and speculated that it was correlated with the higher increase of LASP1 in SK-MES-1 byLASP1 pcDNA (Figure 2).

## 4. Discussion

TGF-*β*1-induced EMT is the initiative and sustained step and plays a central role in the metastasis of lung cancer [29]. The correlation of LASP1 with TGF-*β*1-induced EMT has been firstly identified by Wang et al. [17] in colorectal carcinomas. However, in lung cancer, little is explored about the correlation between LASP1 andTGF-*β*1-induced EMT, although it is observed that LASP1 promotes proliferation, migration, and invasion of NSCLC cell lines [6, 30]. Furthermore, the mechanism of signal transduction from the cytoplasm to the nucleus still needs exploration.

In our study, it was found that TGF-*β*1 upregulates LASP1expression and LASP1 in turn affects the levels of the downstream and key molecules of the TGF-*β*1 signaling pathway both in whole cells (various Smad subtypes) and nuclei (pSmad2, pSmad3, and Snail1). The lung cancer cells were developed EMT by TGF-*β*1 accompanying LASP1 silence or overexpression using LASP1 shRNA lentivirus or LASP1 pcDNA, respectively. *In vitro* results reflected that LASP1 knockdown decreases the invasion, migration, and proliferation of TGF-*β*1-treated cells, while LASP1 overexpression further worsens the malignant behaviors of lung cancer cells. As LASP1 could locate on focal adhesions (podosomes [31] and invadopodia [32]), the leading edges of lamellipodia (pseudopodia), and tips of filopodia, the cellular location hints the invasion and metastasis roles of LASP1 in lung cancer. Beyond LASP1 regulation on the pathological characteristics under TGF-*β*1 stimulation, we also further observed the significant changes of EMT-related markers. After blocking LASP1 expression, EMT hallmarks presented the reverting effects on the deteriorating EMT profiling. LASP1 knockdown increased E-cadherin and decreased N-cadherin and vimentin, while for LASP1 overexpression, they were just the opposite. Taken together, the data in this study reveal that LASP1 might play a regulatory role in the TGF-*β*1-induced EMT process and might be a drug target upon EMT, affecting the initial and sustained stages in the metastasis of lung cancer cells.

TGF-*β*1 canonical signaling is brought about through receptor-regulated Smad (R-Smad), which elicits transcriptional response by binding to Smad Binding Elements (SBE) in cell nuclei and then to repress the epithelial genes expression [33, 34]. Because Smad2 and Smad3 are the most active members in the TGF-*β*1-driven R-Smad family, we especially explored the changes of these two signal molecules. LASP1 silence and overexpression downregulated or upregulated pSmad2 and pSmad3, respectively, not total proteins of Smad2 and Smad3, which showed the effect on the activity of TGF-*β*1 canonical signaling by LASP1. The data reveal that LASP1 plays a role in promoting the phosphorylation of Smad2 and Smad3. Furthermore, it is very complicated for the biological function and mechanisms of TGF-*β*1 in tumor cells. Recently, the inhibitory Smad (I-Smad) has attracted increasing attention as a negative regulator in the TGF-*β*1 pathway. Smad7 is the I-Smad and inhibits TGF-*β*1 signaling by multiple mechanisms, such as competing with R-Smad for receptor binding to repress TGF-*β*1 signaling [35]. Smad7 notably rises in response to activation of TGF-*β*1 signaling, takes inhibitive response to TGF-*β*1 signaling, and abrogates the effects of TGF-*β*1, including EMT. The downregulation of Smad7 promotes lung cancer metastasis under TGF-*β*1 signaling activation [36]. The results in this study showed that LASP1 knockdown decreased Smad7 level, whereas overexpression increased Smad7, which might be that the changed amount of “bad protein” (LASP1) in upstream leads to the subsequent change of Smad7 production in downstream. Because the aggregation of Smad2/3 or Smad7 to TGF-*β*1 receptor is involved in the activation or inhibition of TGF-*β*1 signaling, therefore, whether LASP1 could promote TGF-*β*1 receptor recruit of Smad proteins needs further research.

TGF-*β*1 signaling is known as a dynamic process transmitting signal from the cell membrane and cytoplasm to cell nuclei. Phosphorylation of R-Smad proteins initiates TGF-*β*1 signaling and promotes the dimerization of pSmad2 and pSmad3, followed by their translocation into nuclei [37]. Our results showed the nuclear levels of pSmad2 and pSmad3 were regulated by LASP1under TGF-*β*1 treatment. LASP1 knockdown downregulated the nuclear levels of pSmads proteins so as to inhibit the step of EMT from the cytoplasm into cell nuclei. Also, LASP1 overexpression promoted the EMT through increasing nuclear levels of pSmad2 and pSmad3. Moreover, Snail1, located in the cell nucleus, is the downstream of the TGF-*β*1/Smad/Snail signaling pathway and is required for TGF-*β*1-induced EMT transition through action of Smad2, Smad3, and Smad4 [38]. Snail1 translocates into the cell nucleus to bind to the E-cadherin promoter and represses the transcription of E-cadherin and then to lead to E-cadherin loss in EMT hallmarks. In this study, nuclear Snail1 declined profoundly by LASP1 silence and was increased by LASP1 overexpression. This result coincided with the changed trend of E-cadherin by LASP1. Taken together, the study indicated that LASP1 knockdown could impact multiple levels of TGF-*β*1 signaling, as well as several key molecule expressions in the cell nucleus.

Overall, this study identified the integral changes of TGF-*β*1-mediated EMT by LASP1, from signaling activation and nuclear expression of key signal molecules to EMT-related markers and biological functions in lung cancer cell lines *in vitro*. The biological actions and molecular mechanisms of LASP1 may involve (1) phosphorylation of Smad2 and Smad3; (2) increased protein level of Smad7; (3) upregulated nuclear levels of Snail1, pSmad2, and pSmad3; and (4) deteriorative changes of EMT hallmark proteins (E-cadherin, N-cadherin, and vimentin); as well as (5) enhanced pathological features, such as invasion, migration, and proliferation. These results suggested that LASP1 could regulate TGF-*β*1-induced EMT by regulating the Smad and Snail1 signal.

## Figures and Tables

**Table 1:** Average optical density values of LASP1 protein expressions by immunohistochemistry staining in human nonmalignant adjacent tissues and lung adenocarcinoma tissues (*n* = 12).

Group	Average optical density (×10^2^)
Adjacent tissues	0.84 ± 0.29
Lung adenocarcinoma tissues	3.32 ± 0.38^∗∗∗^

^*∗∗∗*^ *p* < 0.001.

## Data Availability

The data used to support the findings of this study are included within the article.

## Supplementary Materials

Supplementary Materials This section provides additional information about the siRNA target sequence (Supplementary Table S1), knockdown efficiency (Supplementary Figure S1), and the cell proliferation by TGF-*β*1, knockdown, or overexpression treatment (Supplementary Figure S2).
