# Supplementary material for: LASP1 Induces Epithelial-Mesenchymal Transition in Lung Cancer through the TGF-β1/Smad/Snail Pathway
Source: Can Respir J. 2021 Dec 6;2021:5277409. doi: 10.1155/2021/5277409 (PMC8668282; doi:10.1155/2021/5277409)

**Supplementary Data**

Figure S1. LASP1 knockdown efficiency in A549 cells by quantitative real-time PCR analysis. A549 cells were transfected with scramble siRNA (SC siRNA), siRNA-1, -2, -3 against LASP1 for 48 h. The siRNA-mediated gene knockdown efficiencies for LASP1 were analyzed by quantitative real-time PCR. All quantitative amounts were normalized against GAPDH expression and shown as mean ± SD, n=3. ^***^P＜0.001 *vs.* A549 cells alone group.


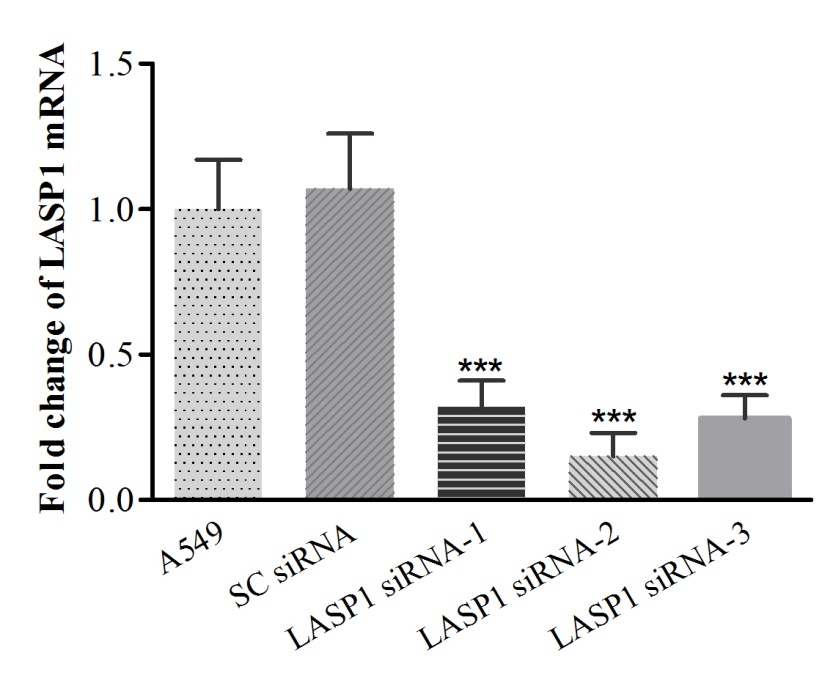


**Table.S1.** Target mRNA sequences of LASP1 siRNA duplexes

|  | siRNA target sequence |
| --- | --- |
| LASP1 siRNA duplex 1 | GCGCTACAAGGAGGAGTTTGAGA |
| LASP1 siRNA duplex 2 | AAGGTGAACTGTCTGGATAAG |
| LASP1 siRNA duplex 3 | CAGGTTCTTGCCTTTCTTAATTT |

Figure S2. Effect of LASP1 on A549 and SK-MES-1 cells proliferation in the presence or absence of TGF-β1 treatment. LASP1 silence or overexpression took effect on A549 cells for 48 h treatment (a) or 72 h treatment (b). LASP1 silence or overexpression took effect on SK-MES-1cells for 48 h treatment (c) or 72 h treatment (d). Data was shown as mean ± SD, n=6. ^*^P＜0.05 and ^**^P＜0.01 *vs.* cell alone group without TGF-β1 treatment, ^#^P ＜ 0.05, ^##^P ＜ 0.01 and ^###^P ＜ 0.001 *vs.* TGF-β1-treated non-infection group.


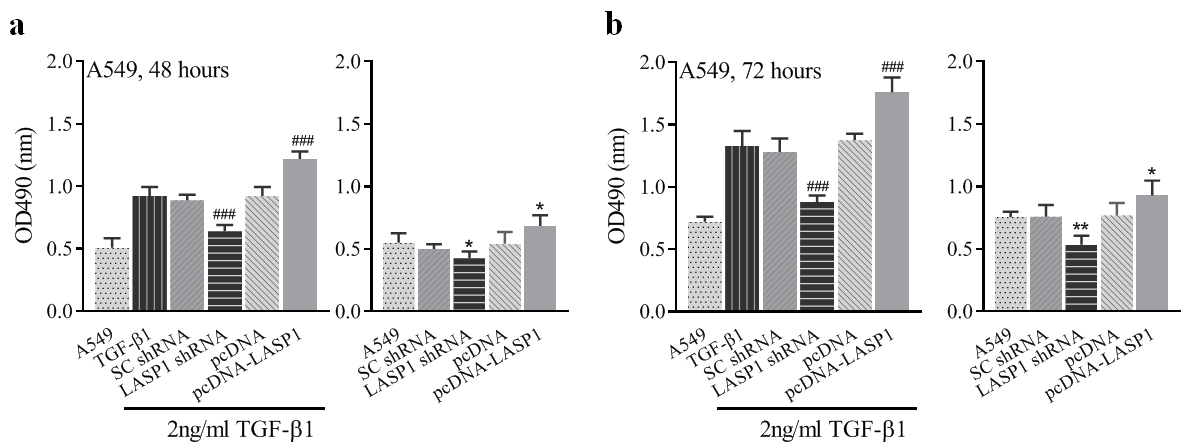


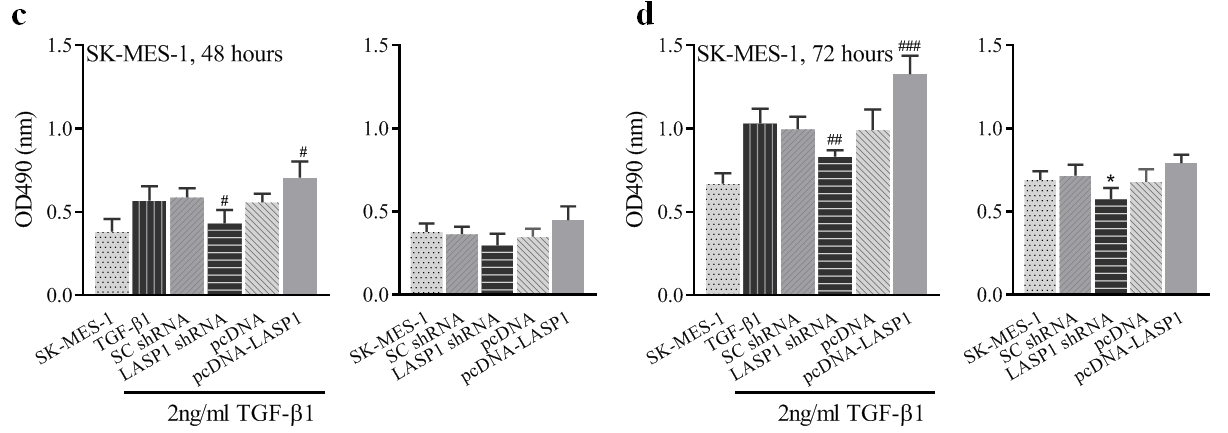

Supplement: Supplementary Materials — This section provides additional information about the siRNA target sequence (Supplementary Table S1), knockdown efficiency (Supplementary Figure S1), and the cell proliferation by TGF-β1, knockdown, or overexpression treatment (Supplementary Figure S2). [file 5277409.f1.docx]
